# Perceived benefits and constraints in vehicle automation: Data to assess the relationship between driver's features and their attitudes towards autonomous vehicles

**DOI:** 10.1016/j.dib.2019.104662

**Published:** 2019-10-16

**Authors:** Ignacio Lijarcio, Sergio A. Useche, Javier Llamazares, Luis Montoro

**Affiliations:** a INTRAS (Research Institute on Traffic and Road Safety), University of Valencia, Spain; b Spanish Foundation for Road Safety (FESVIAL), Spain; c Department of Technology, ESIC Business and Marketing School, Spain

**Keywords:** Spanish drivers, Autonomous vehicles, Attitudes, Perception, Intention, Road Safety

## Abstract

This data article examines the association driver's features, perceptions and attitudes towards autonomous vehicles (AVs). The data was collected using a structured self-administrable and online-based questionnaire, applied to a full sample of 1205 Spanish drivers. The data contains 4 parts: the full set of bivariate correlations between study variables; descriptive statistics and graphical trends for each main study variable according to gender, age group and city/town size; and, finally, the dataset for further explorations in this regard. For more information, it is convenient to read the full article entitled “*Perceived safety and attributed value as predictors of the intention to use autonomous vehicles: A national study with Spanish drivers*” [1].

**Table:** Specifications Table

Subject area	*Psychology*
More specific subject area	*Autonomous Vehicles; Spanish Drivers; Demographics; Acceptance; Attitudes; Road Safety.*
Type of data	*Tables, graphs, database*
How data was acquired	*Original data was collected through a national web-based survey. The questionnaire is available as supplementary material of this data article. The data was consolidated and analyzed through the statistical software package IBM SPSS (version 24.0) for descriptive procedures and IBM SPSS AMOS (version 24.0) for structural/inferential ones*
Data format	*Raw, filtered and analyzed*
Experimental factors	*Population consisted of a sample of Spanish drivers, about which their perceptions and valuations on the autonomous vehicles (AVs) were analyzed*
Experimental features	*Study of user profile-based differences on the acceptance and attitudes towards AVs through comparative and graphical analyses*
Data source location	*Europe*
Data accessibility	*Data is with this article*
Related research article	*Montoro* L*, Useche S, Alonso F, Lijarcio J, Bosó-Seguí P., Martí-Belda A. Perceived safety and attributed value as predictors of the intention to use autonomous vehicles: A national study with Spanish drivers. Saf Sci. 120C (2019).*https://doi.org/10.1016/j.ssci.2019.07*.041*

**Table:** 

**Value of the Data** • This data can be useful since it provides information on how Spanish drivers perceive the safety and value of the autonomous vehicles (AVs), and their intention to use them. • This data can be used by other researchers, road safety practitioners and market stakeholders to identify demographic-based patterns and profiles of potential users of AVs, according to the trends and differences identified in this study. • The perceived safety and value attributed to autonomous vehicles can be analyzed according to different user-related features, such as their age, gender, educational level, city/town size and occupation, variables also contained in the annex dataset. • Additionally, the data contained in this article can be compared with other samples/studies, in order to examine means, associations and trends on perceptions and attitudes towards the autonomous vehicles (AVs) among drivers.

## 1 Data

**Fig. 1:**
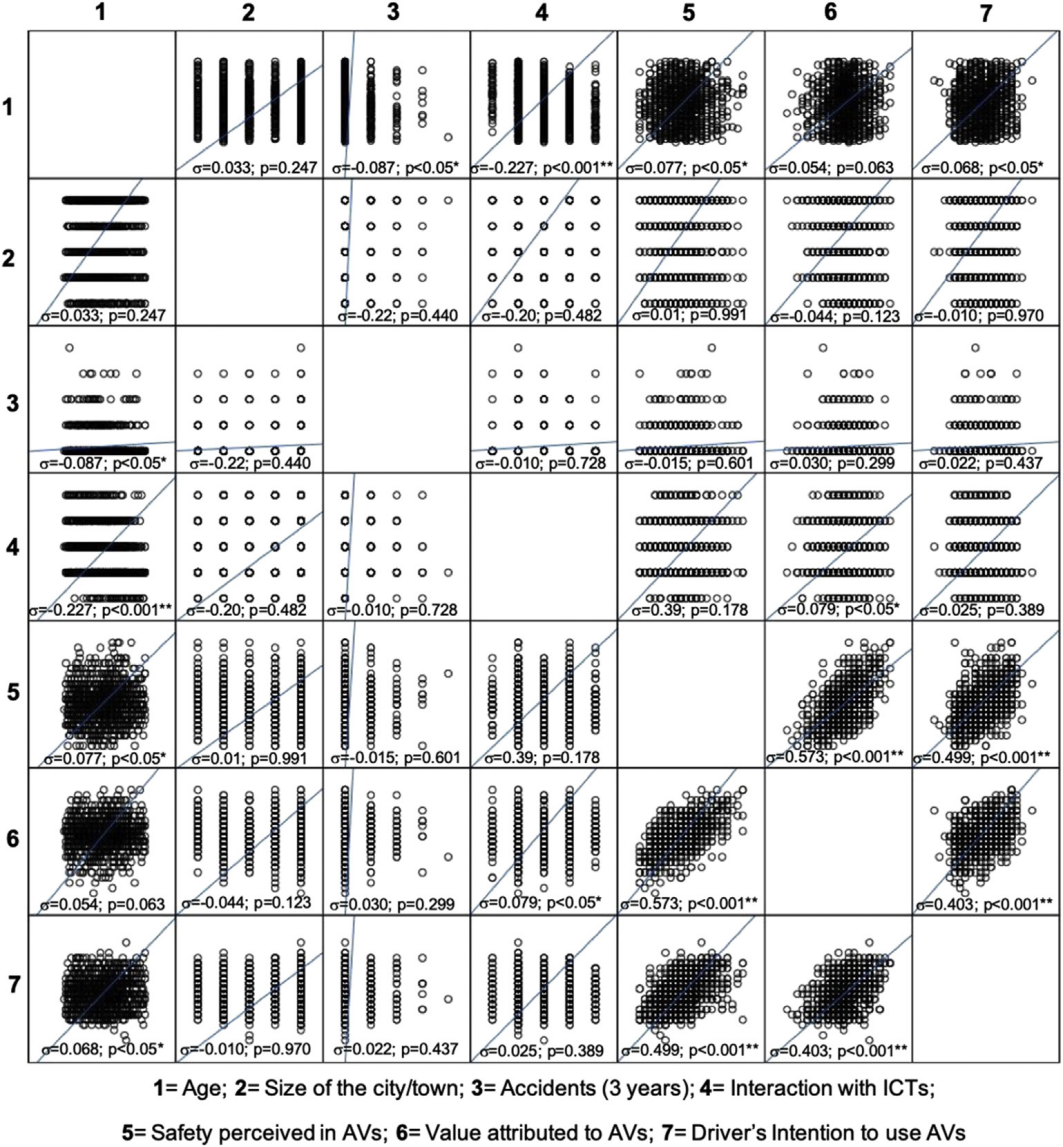
Bivariate correlations between study variables (demographics, driving issues and AV-related perceptions) among Spanish drivers.

The dataset of this article provides information on a set of demographics, perceptions on autonomous vehicles and crash-related factors of the sample, fully composed of licensed Spanish drivers. Table 1 presents the descriptive information on the items contained in the questionnaire. Fig. 1 presents graphically the full set of bivariate correlations among the three main study factors and individual features of drivers.

**Table 1:** Descriptive statistics of AV-related study variables (factors) contained in the data set and gender-based differences.

Items in the questionnaire	N^1^	Min^2^	Max^3^	Mean^4^	SD^5^
Factor 1: Perceived Safety (5 items; α = 0.735)					
1. Overall. AVs would help make my journeys safer than they are when I use conventional cars	1205	1	5	3.29	1.00
2. AVs would act better than myself in a complicated traffic situation	1205	1	5	2.95	.99
3. A driverless/automated vehicle may be not “smart” enough for guaranteeing my safety during the journey (−)	1205	1	5	3.79	1.00
4. AV-related systems could easily break down, or be hacked, thus compromising my safety (−)	1205	1	5	3.81	1.03
5. AVs would respond adequately to unexpected situations that commonly require rapid responses from drivers	1205	1	5	2.62	1.20
Factor 2: Value Attributed (5 items; α = 0.739)					
1. They would help improve the traffic flow, making journeys more agile and efficient	1205	1	5	3.22	1.05
2. They would reduce fuel use and improve the environment	1205	1	5	3.46	.99
3. They might contribute to reduce crashes and injuries caused by traffic accidents	1205	1	5	3.22	1.04
4. I believe the cost-benefit relation of AVs would not be balanced, and costs might overcome the benefits (−)	1205	1	5	3.88	1.00
5. They would contribute to reducing the misbehaviors of drivers, and to strengthening respect and co-existence on the road	1205	1	5	4.22	.95
Factor 3: Intention to Use (5 items; α = 0.929)					
1. I would prefer using an AV more than a conventional car when driving on urban/city roads	1205	1	5	2.63	1.33
2. If during the next years I will have enough budget, I plan to buy an AV	1205	1	5	2.41	1.27
3. I would prefer using an AV than a conventional car if I were tired	1205	1	5	3.72	1.31
4. I am totally against the option of buying an autonomous car (−)	1205	1	5	2.61	1.36
5. Considering the need of adapting to transport dynamics, planning to buy an AVs at some point in the next years sounds adequate	1205	1	5	2.77	.98

*Notes:* Negative (-) items have been recoded for factor scoring.; ^1^n= sample size; ^2^Min= lower value; ^3^Max= higher value; ^4^Mean*=* Arithmetic mean (*average*); ^5^SD= Standard Deviation.

**Fig. 2:**
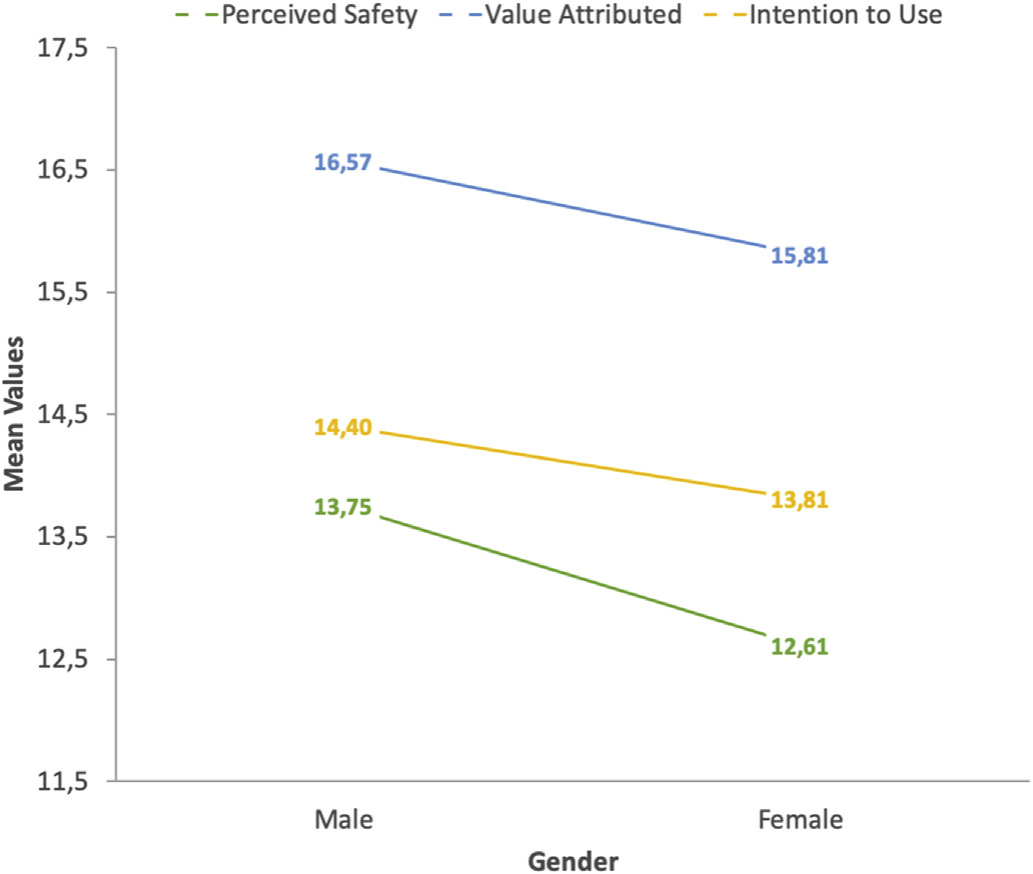
Gender-based trends in the autonomous vehicle’ appraisal of Spanish drivers.

**Fig. 3:**
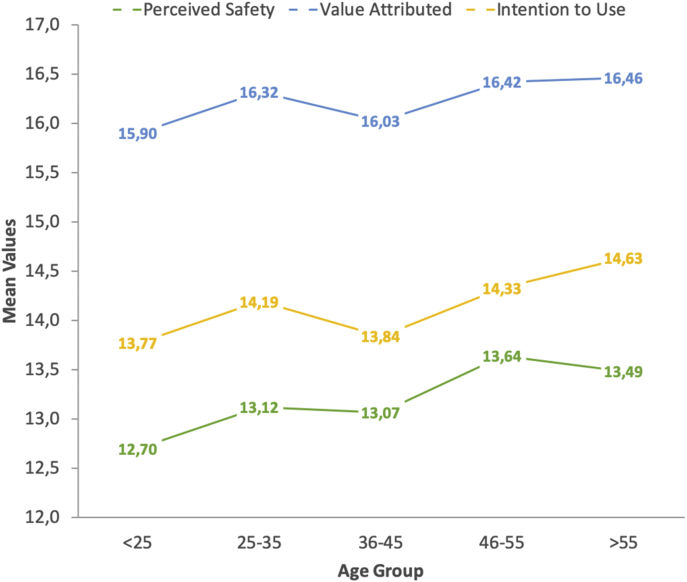
Gender-based trends on the autonomous vehicle’ appraisal (safety, value and intention).

Table 2 shows the descriptive statistics obtained for the three attitudinal AV-related variables included in this data article (i.e., perceived safety, value attributed and intention to use), both for the full sample and split by gender, and Fig. 2 specifically shows trends on acceptance of autonomous vehicles according to the gender of drivers. Table 3 allows to identify the specific differences between drivers by age through a One-way ANOVA (Analysis of Variance), summarizing the statistical differences among different age groups for these variables, and Fig. 3 graphically shows the mean score reported by each age group.

**Table 2:** Descriptive statistics of AV-related study variables (factors) contained in the data set and gender-based differences.

Variable	Gender	N	Mean	SD^1^	SE^2^	95% CI^3^		ANOVA		
						Lower	Upper	F	*p*	Sig.
Perceived Safety	Female	538	12.62	3.20	.14	12.35	12.89	32.665	<.001	**
	Male	667	13.75	3.59	.14	13.48	14.03			
	Total	1205	13.25	3.47	.10	13.05	13.44			
Value Attributed to Autonomous Vehicles	Female	538	15.81	2.62	.11	15.59	16.03	21.685	<.001	**
	Male	667	16.58	3.00	.12	16.35	16.80			
	Total	1205	16.24	2.86	.08	16.07	16.40			
Intention to Use an Autonomous Car	Female	538	13.82	2.97	.13	13.56	14.07	11.194	.001	*
	Male	667	14.39	2.95	.11	14.17	14.61			
	Total	1205	14.13	2.97	.09	13.97	14.30			

*Notes:*^*1*^*SD* = Standard Deviation; ^*2*^*SE* = Standard Error; ^*3*^*95% CI* = Confidence Interval at the level 95%; *Significant at the level 0.05; **Significant at the level 0.01.

**Table 3:** Age-based differences in perceptions on the autonomous vehicle among Spanish drivers.

Variable	Gender	N	Mean	SD^1^	SE^2^	95% CI^3^		ANOVA		
						Lower	Upper	F	*p*	Sig.
Perceived Safety	<25	113	12.70	3.15	0.30	12.11	13.29	2.49	.050	*
	25–35	271	13.12	3.53	0.21	12.70	13.54			
	36–45	359	13.07	3.35	0.18	12.72	13.41			
	46–55	326	13.64	3.69	0.20	13.24	14.04			
	>55	136	13.49	3.30	0.28	12.93	14.04			
	Total	1205	13.25	3.47	0.10	13.05	13.44			
Value Attributed to AVs	<25	113	15.90	2.91	0.27	15.36	16.45	1.47	.210	^N/S^
	25–35	271	16.32	2.93	0.18	15.97	16.67			
	36–45	359	16.03	2.91	0.15	15.72	16.33			
	46–55	326	16.42	2.73	0.15	16.12	16.72			
	>55	136	16.46	2.83	0.24	15.98	16.94			
	Total	1205	16.24	2.86	0.08	16.07	16.40			
Intention to Use AVs	<25	113	13.77	2.73	0.26	13.26	14.28	2.68	.031	*
	25–35	271	14.19	2.98	0.18	13.84	14.55			
	36–45	359	13.84	2.91	0.15	13.54	14.14			
	46–55	326	14.33	3.01	0.17	14.00	14.66			
	>55	136	14.63	3.14	0.27	14.10	15.16			
	Total	1205	14.13	2.97	0.09	13.97	14.30			

*Notes:*^*1*^*SD* = Standard Deviation; ^*2*^*SE* = Standard Error; ^*3*^*95% CI* = Confidence Interval at the level 95%; *Significant at the level 0.05.

Finally, Table 4 presents the mean scores reported on perceived safety, value attributed and intention to use autonomous vehicles (AVs) according to the size of the town/city of residence of participants. In addition, this article includes, as supplementary materials: the questionnaire (form) used for performing the study, and the dataset (SPSS -.sav), that will allow researchers to perform additional tests and comparisons using the entire set of measured variables. It is important to remark that no inferences, interpretations or conclusions on the data are presented in this paper, but are available in the complementary article [1].

**Table 4:** Town-size-based differences for main study variables.

Variable	Gender	N	Mean	SD^1^	SE^2^	95% CI^3^		ANOVA		
						Lower	Upper	F	*p*	Sig.
Perceived Safety	<5000	96	13.69	3.71	0.38	12.94	14.44	0.798	.525	^N/S^
	5000−0.2 M	208	12.97	3.57	0.25	12.48	13.46			
	0.2 M−0.5 M	172	13.31	3.34	0.25	12.81	13.81			
	0.5 M−1 M	145	13.12	3.85	0.32	12.49	13.75			
	>1 M	584	13.29	3.33	0.14	13.02	13.56			
	Total	1205	13.25	3.47	0.10	13.05	13.44			
Value Attributed to AVs	<5000	96	17.23	2.67	0.27	16.69	17.77	3.307	.010	*
	5000−0.2 M	208	16.03	2.79	0.19	15.65	16.41			
	0.2 M−0.5 M	172	16.13	2.72	0.21	15.72	16.54			
	0.5 M−1 M	145	16.22	3.34	0.28	15.67	16.77			
	>1 M	584	16.18	2.80	0.12	15.95	16.41			
	Total	1205	16.24	2.86	0.08	16.07	16.40			
Intention to Use AVs	<5000	96	14.94	2.87	0.29	14.36	15.52	3.205	.013	*
	5000−0.2 M	208	13.66	3.02	0.21	13.25	14.08			
	0.2 M−0.5 M	172	14.05	2.96	0.23	13.60	14.49			
	0.5 M−1 M	145	14.27	3.01	0.25	13.78	14.76			
	>1 M	584	14.16	2.95	0.12	13.92	14.40			
	Total	1205	14.13	2.97	0.09	13.97	14.30			

*Notes:*^1^SD = Standard Deviation; ^2^SE = Standard Error; ^3^95% CI = Confidence Interval at the level 95%; *Significant at the level 0.05.

## 2 Experimental design, materials, and methods

### 2.1 Participants

For this cross-sectional research, it was collected and analyzed the data of a nationally representative sample of *n* = 1025 drivers from the 17 autonomous communities of Spain.

In accordance with the pursued analyses and some previous research experiences dealing with different gender and age-based groups of population [2,3], the data was analyzed considering both the full sample and specific sub-groups built up bearing in mind these individual features, already supported by literature as potential key factors on decision-making in urban mobility-settings [[4], [5], [6]]. Thus, for making comparisons in the study variables, the full sample was divided: *a*) by gender (538 females, and 667 males); and *b*) in five intervals, composed as follows: <25 years (*n =* 113, composing 9.4% of the sample); 25–35 years (*n =* 271, composing 22.4% of the sample); 36–45 years (*n =* 359, composing 29.8% of the sample); 46–55 years (*n =* 326, composing 27.1% of the sample); and >55 years (*n =* 136, composing 11.3% of the sample). Additionally, it was taken into account the size of the town/city of residence of the driver, as recent evidences suggest that attitudes towards autonomous vehicles may differ according to the place of residence [6] and other settings related to driving habits and lifestyle [3,4].

### 2.2 Questionnaire

For the original research [1], the questionnaire was administrated exclusively in Spanish (professionally translated for publication) and consisted of three main sections. The first part asked about individual and demographic variables, such as age, gender, city/town of provenance (and its size) and main current occupation.

In the second part, participants were asked about their level of interaction with Information and Communication Technologies (ICTs) in a scale between 1 (less interaction) and 5 (more interaction). It also contained a short form about driving-related issues such as: crashes suffered while driving conventional cars (along the last 3 years), driving tenure (years licensed) and driving patterns, including the type of vehicle most frequently driven, number of kilometers (Km) a day, and their average frequency (times a week), in order to estimate the driving intensity.

As for the third part of the research questionnaire, a 5-item scale (*α =* 0.735) was used for measuring the perceived safety of autonomous vehicles among drivers. It asked the level of agreement of drivers with statements related to the safety features of AVs through a Likert scale between 1 = total disagreement to 5 = total agreement. In order to assess the value attributed to the AV for traffic sustainability and road safety, it was applied a 5-item scale (*α =* 0.739), aimed at obtaining the appraisal of participants on topics related to the impact of AVs on better and safer transport dynamics, using a Likert form ranging from 1 to 5. Finally, and in order to measure the intention of using autonomous vehicles, a 12-item (*α =* 0.929) Likert scale (1 = total disagreement to 5 = total agreement), designed under the guidelines suggested by Van Der Laan et al. [7] was applied. It asked participants about different situations in which they would decide (or not) to use an autonomous vehicle, considering the potential benefits seen on it by them. The full set of items included in the questionnaire and their descriptive statistics are shown in Table 1.

### 2.3 Statistical analysis

First of all, basic descriptive analyses (i.e. means and standard deviations of the study variables) were obtained, and bivariate correlation analyses were carried out, in order to establish measures of association between pairs of these factors. Further, and with the aim of comparing the scores obtained on attitudes towards autonomous vehicles, One-way ANOVA (Analysis of Variance) was performed for the categorical factors: 1) gender; 2) age group - using five intervals, as described in the sample section; and 3) width of the city of provenance. The full set of variables and cases composing the study is available in the annex dataset.
