# Supplementary material for: Perceived benefits and constraints in vehicle automation: Data to assess the relationship between driver's features and their attitudes towards autonomous vehicles
Source: Data Brief. 2019 Oct 16;27:104662. doi: 10.1016/j.dib.2019.104662 (PMC6838434; doi:10.1016/j.dib.2019.104662)
Supplement: Multimedia component 1 [file mmc1.docx]

**Supplementary material**

**Questionnaire contents (root - researcher form)**

Questionnaire code: _________

**Section I.**

*Please answer the following questions. Remember that in no case we will ask for your name, or for any personal data that could identify you.*

Age: _____

Gender: _________

City or town of residence: ___________________________________

Size (population) of your city/town of residence:

- <5,000 inhabitants
- Between 5,000-20,000 inhabitants
- Between 20,000-50,000 inhabitants
- Between 50,000-100,000 inhabitants
- >100,000 inhabitants

Highest academic level (completed):

- None (no studies)
- Primary studies
- Secondary studies / high school
- Technical studies
- University studies

Current occupation: ________________________________________

**Section II.**

*In this section, we will ask some questions about your driving habits and other related variables. Please tell us:*

For how long (years) have you had your driver license?: __________ years.

Note: if you do not own a driver license, please finish the survey here.

What type of vehicle do you drive the most, meaning routinely?:

- Private car
- Motorcycle/moped/two-wheeled
- Van
- Heavy vehicle (truck, bus, freight)
- Other: _________________.

Approximately, how many days per week do you drive? (0-7): __________.

Note: if you drive less than once per month, please finish the survey here.

How many kilometers do you drive daily, considering the sum of all your usual journeys?:

- <10 Km/day
- Between 10-30 Km/day
- Between 31-70 Km/day
- >70 Km/day

Regardless of their severity, how many traffic accidents have you suffered during the past three (3) years?: ______

**Interaction with ICTs –** Please tell us your degree of usual interaction with this type of technology (cellphones, computers and/or smart devices which are normally connected to the Internet). Below the question you will find some criteria that will help you answer.

Very scarce

Scarce

Mid

High

Very high

**Very scarce**: No use of these devices, except in some specific cases in which it is absolutely necessary.

**Scarce**: My interaction with this type of devices could be considered low. I use them only when necessary.

**Mid**: I sometimes interact with this type of devices, but not with a regular frequency.

**High**: I use them very frequently, but not necessarily every day nor for most of my activities.

**Very high**: I interact with technological devices on a daily basis, and I use them for almost all of my activities.

**Section III.**

**Perceived safety -** *In this section, we will mention some issues related to how safe Autonomous Vehicles (AVs) are, according to your perception. Please, state to what extent you agree with the following statements:*

Totally disagree – Disagree – Neutral – Agree – Totally agree

1. Overall, AVs would help make my journeys safer than they are when I use conventional cars
2. AVs would act better than myself in a complicated traffic situation
3. A driverless/automated vehicle may be not “smart” enough for guaranteeing my safety during the journey (-)
4. AV-related systems could easily break down, or be hacked, thus compromising my safety (-)
5. AVs would respond adequately to unexpected situations that commonly require rapid responses from drivers

**Value -** *Below, we will address some potential improvements in different spheres that AVs may (or not) imply. Please, state to what extent you agree with the following statements:*

Totally disagree – Disagree – Neutral – Agree – Totally agree

1. They would help improve the traffic flow, making journeys more agile and efficient
2. They would reduce fuel use and improve the environment
3. They might contribute to reduce crashes and injuries caused by traffic accidents
4. I believe the cost-benefit relation of AVs would not be balanced, and costs might overcome the benefits (-)
5. They would contribute to reducing the misbehaviors of drivers, and to strengthening respect and co-existence on the road

**Intention –** *Finally, we would like to present some brief statements about your intention of using AVs instead conventional vehicles:*

Totally disagree – Disagree – Neutral – Agree – Totally agree

1. I would prefer using an AV more than a conventional car when driving on urban/city roads
2. If during the next years I will have enough budget, I plan to buy an AV
3. I would prefer using an AV than a conventional car if I were tired
4. I am totally against the option of buying an autonomous car (-)
5. Considering the need of adapting to transport dynamics, planning to buy an AVs at some point in the next years sounds adequate
